# Anti-BCL2 therapy eliminates giant congenital melanocytic nevus by senolytic and immune induction

**DOI:** 10.1038/s41392-025-02247-2

**Published:** 2025-05-16

**Authors:** Boxuan Wei, Qingxiong Yu, Jiamin Jin, Danli Zhu, Bohan Lai, Jieyu Gu, Ran Yang, Huailiang Huang, Hongzhan Lin, Liang Zhang, Tao Zan, Feng Xie, Kang Zhang, Qingfeng Li

**Affiliations:** 1 Department of Plastic and Reconstructive Surgery, Shanghai Ninth People’s Hospital, Shanghai Jiao Tong University School of Medicine, Shanghai, China; 2 Department of Facial Plastic and Reconstructive Surgery, Eye & ENT Hospital of Fudan University, Shanghai, China; 3 CAS Key Laboratory of Tissue Microenvironment and Tumor, Shanghai Institute of Nutrition and Health, University of Chinese Academy of Sciences, Chinese Academy of Sciences, Shanghai, China; 4 Shanghai Key Laboratory of Reproductive Medicine, Shanghai, China; 5 Center for Biomedicine and Innovations, Faculty of Medicine, Macau University of Science and Technology, Macau, China; 6 Eye Hospital and Institute for Advanced Study on Eye Health and Diseases, Wenzhou Medical University, Wenzhou, China; 7 Zhuhai International Eye Center, Zhuhai People’s Hospital and the First Affiliated Hospital of Faculty of Medicine, Macau University of Science and Technology, Guangdong, China

**Keywords:** Paediatric cancer, Skin cancer, Translational research, Target identification

## Abstract

Giant congenital melanocytic nevus (GCMN) is a RAS/RAF mutation-driven syndrome characterized by extensive melanocytic lesions, posing psychological challenges and a lifelong risk of malignancy. Existing treatments like surgical resection and laser therapy fail to fully remove lesions, and MAPK inhibitors show limited efficacy. This study identified a predominant population of senescent cells and a minority of proliferative cells in GCMN, necessitating dual-targeted strategies. We found that the anti-apoptotic protein BCL2 is expressed in both senescent and proliferative cells from GCMN patients with various gene mutations. Coexpression of P16 and BCL2 indicated a phenotype of growth arrest and cell survival. BCL2 inhibitors (BCL2i) showed significant cytotoxicity to GCMN cells in vitro. Hypopigmentation and GCMN cell clearance were observed in patient-derived xenograft models and in *Nras*^Q61K^-mutated and *Braf*^V600E^-mutated transgenic models following BCL2i treatment. Histology of regressed GCMN indicated extensive immune cell infiltration, suggesting immune involvement. Single-cell sequencing and immunostaining revealed that activated neutrophils formed extracellular traps, synergizing with BCL2i to treat GCMN. Neutrophil depletion and immunosuppression reduce treatment efficacy, highlighting the crucial role of the immune response post-BCL2i treatment. Long-term follow-up showed no recurrence, with neutrophils and T cells residing in the dermis, indicating memory immune reactions. These findings present a promising therapeutic strategy and underscore the translational potential of BCL2i in treating GCMN.

## Introduction

Giant congenital melanocytic nevus (GCMN) is a benign skin disorder characterized by extensive pigmented lesions present at birth. GCMN arises from the abnormal proliferation of melanocytes during fetal development and is estimated to occur in 1/20000 of newborns.^1,2^ GCMN poses life-threatening risks, with approximately 3–8% of cases progressing to malignant melanoma^3–5^ and 5–8% progressing to neurocutaneous melanocytosis.^6,7^ Beyond these risks, the disfiguring appearance and severe pruritus associated with GCMN can profoundly impair patients’ quality of life and increase their susceptibility to mental health disorders.^8,9^ Current primary treatments, including surgical excision,^10^ dermabrasion, and laser therapy,^11^ often fail to completely remove GCMN lesions, particularly those larger than 60 cm in diameter, and may result in extensive scarring or recurrence. Therefore, the development of effective pharmacotherapies for clearing GCMN lesions is imperative.

Somatic mutations in *NRAS* (68%), *BRAF* (7%),^12^ and *RAF* fusion (5%)^13^ are pivotal drivers of GCMN, activating the MAPK and PI3K-AKT pathways that lead to nevus cell formation. Previous studies have demonstrated the efficacy of inhibitors targeting these pathways in managing GCMN both in vitro and in vivo.^14–17^ Moreover, MEK inhibitors have been utilized to alleviate severe symptoms such as pruritus, pain, swelling, and tissue bulk in GCMN^13,18,19^; however, complete regression of GCMN lesions has not been achieved with MEK inhibitors, indicating the survival of a large number of GCMN cells in the skin posttreatment. This therapeutic limitation implies that GCMN cell survival may not depend solely on proliferative signaling pathways. Clinically, darkening or enlargement of the lesions is rarely observed in never-treated GCMNs according to the previous study^20^ and our observations, suggesting that GCMN may have entered a state of growth arrest rather than active proliferation. Elevated expression of cell cycle inhibitors such as P16, P21, and P53 has been noted in *NRAS-*mutated and *BRAF*-mutated tumors, providing negative feedback to halt proliferation and prevent malignancy.^21,22^ Similarly, GCMN lesions exhibit high P16 expression and extremely low Ki67 expression,^23^ indicating that this protective process may be activated in a large proportion of GCMN cells. In addition to inducing cell cycle arrest, P16 overexpression has been reported to be lethal to cells^24^; however, most GCMN cells persist in the patient’s body for their entire life, prompting investigations into their mechanisms of prolonged survival.

In this study, we detected robust antiapoptotic activity and widespread BCL2 expression in GCMN via RNA sequencing (RNA-seq) and immunostaining of tissue microarrays. Double immunostaining revealed that most GCMN cells in the superficial and deep dermis strongly coexpressed P16 and BCL2. These findings indicate a phenotype of “growth arrest and anti-apoptosis” in GCMN cells, akin to the characteristics of senescent cells. In the current study, various senescence markers were investigated in GCMN cells, and the results revealed that these cells exhibited the senescence-associated secretory phenotype (SASP), expressed senescence-associated heterochromatin foci (SAHF), and lost Lamin B1, distinct features not typically found in normal melanocytes. The predominance of senescent nevus cells within GCMN tissues may explain the limited efficacy of MEK inhibitors, which primarily target proliferative pathways. This observation emphasizes the critical need for therapies that effectively target both proliferative and senescent nevus cells to achieve complete lesion regression.

Senolytic therapies, designed to eliminate senescent cells, have emerged as a promising strategy for treating age-related diseases, fibrosis, and certain tumors.^25,26^ Senescent cells are characterized by their resistance to apoptosis, which allows them to persist in tissues for extended periods. This apoptosis resistance is often mediated by upregulated anti-apoptotic pathways, such as the BCL2 family, PI3K/AKT pathway, and HSP90.^27,28^ Consequently, senolytic agents that target these pathways have shown efficacy in selectively inducing apoptosis in senescent cells while sparing normal cells.^27^ Given that the majority of GCMN cells co-express P16 and BCL2, senolytic therapies targeting BCL2 may offer a viable approach for treating GCMN. Moreover, we discovered that BCL2 is expressed in both P16+ and Ki67+ GCMN cells, indicating its potential as a therapeutic target capable of eliminating both senescent and proliferative nevus cells. Our in vitro and in vivo studies revealed that BCL2 inhibitors were able to clear more nevus cells than MEK inhibitors. Silencing of BCL2 expression in GCMN cells also demonstrated notable efficacy. Transgenic mouse models (H11-*Tyr*-*Nras*^Q61K^ and H11-*Tyr*-*Braf*^V600E^) were developed to replicate GCMN phenotypes, confirming the efficacy of BCL2 inhibitors in inducing nearly complete regression of nevi and associated leukotrichia. Intriguingly, this study revealed a synergistic effect of BCL2 inhibitors on the recruitment and immune response of neutrophils, as evidenced by single-cell sequencing and immunostaining. Depletion of neutrophils in GCMN model mice attenuated approximately 52% of the therapeutic effects of BCL2 inhibitors, underscoring the involvement of immune responses in the treatment of this cutaneous disease. Resident neutrophils prevented the recurrence of GCMN according to the long-term follow-up. In conclusion, our findings suggest that BCL2 inhibitors hold promise as a therapeutic approach for GCMN, eliminating GCMN cells by inducing apoptosis and triggering immune responses. This study addresses current limitations in treatment options and highlights potential avenues for future clinical trials.

## Results

### BCL2 expression and GCMN cell survival

**Fig. 1:**
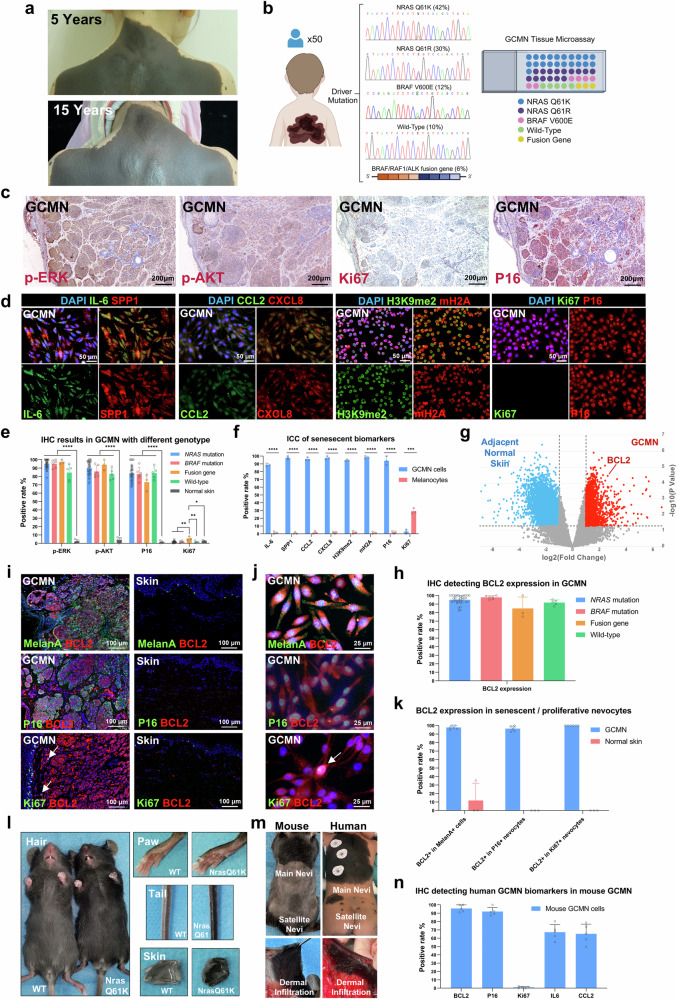
Senescent and anti-apoptotic characteristics detected in human GCMN and mouse GCMN models. **a** Clinical images demonstrating the stable GCMN pattern over different periods in the same patient. **b** Diagram illustrating the driver mutations of 50 included GCMN patients and the arrangement of GCMN tissue microarray. **c** Immunohistochemistry staining (AEC) showing p-ERK, p-AKT, Ki67, and P16 expression in GCMN tissue microarray. Scale bar=200μm. **d** Immunocytochemistry of primary GCMN cells stained for senescence-associated secretory phenotype biomarkers (IL-6, SPP1, CCL2, and CXCL8), senescence-associated heterochromatic foci (H3K9me2 and mH2A), and cell proliferation markers (Ki67 and P16). Scale bar=50μm. **e** Positive rates of immunohistochemistry for different genotypes of GCMN tissues (*n* = 36 patients with *NRAS* mutation, 6 with *BRAF* mutation, 3 with fusion gene, 5 with wild-type genotype) and normal skin tissues (*n* = 5 healthy donors). Mean ± SD. **f** Positive rates of immunocytochemistry detecting senescent biomarkers in GCMN cells (*n* = 3 patients) and melanocytes (*n* = 3 healthy donors). Mean ± SD. **g** Volcano plot showing differentially expressed genes between GCMN and adjacent normal skin tissues according to RNA-seq analysis. Red dots represent upregulated genes in GCMN, while blue dots represent upregulated genes in adjacent normal skin tissues. The expression of the anti-apoptotic gene BCL2 is highlighted. **h** Positive rates of immunohistochemistry detecting BCL2 expression in various GCMN genotypes. (*n* = 36 patients with *NRAS* mutation, 6 with *BRAF* mutation, 3 with fusion gene, 5 with wild-type genotype). Mean ± SD. **i** Immunostaining of MelanA/BCL2, P16/BCL2 and Ki67/BCL2 in GCMN and normal skin. White arrows indicate Ki67 + BCL2 + GCMN cells. Scale bar=100μm. **j** Immunocytochemistry staining of MelanA/BCL2, P16/BCL2 and Ki67/BCL2 in GCMN cells. White arrows indicate Ki67 + BCL2 + GCMN cells. Scale bar=25μm. **k** Positive rates of immunohistochemistry detecting BCL2 expression in MelanA+ cells, P16+ senescent nevocytes and Ki67+ proliferative nevocytes. GCMN (*n* = 6 patients), Normal skin (*n* = 3 healthy donors). Mean ± SD. **l** Phenotypic comparison between H11-*Tyr*-*Nras*^Q61K^ and wild-type (WT) mice. **m** H11-*Tyr*-*Nras*^Q61K^ mice model the phenotypes of human GCMN. **n** Positive rates of immunohistochemistry detecting anti-apoptotic biomarker (BCL2), senescent biomarkers (P16, IL-6, and CCL2), and growth arrest (Ki67) in the GCMN lesions of H11-*Tyr*-*Nras*^Q61K^ mice (*n* = 5 mouse). Mean ± SD

BCL2 expression contributes to the prolonged survival of both P16+ senescent and Ki67+ proliferative giant congenital melanocytic nevus (GCMN) cells. Clinically, GCMN lesions maintain consistent dimensions and proportions over time (Fig. 1a), persisting throughout patients’ lifetimes, which suggests a phenotype of “growth arrest and cell survival” similar to senescence. To investigate this, tissue samples from 50 GCMN patients with various genetic mutations (*NRAS*, *BRAF*, gene fusion, and wild-type) were collected (Fig. 1b) and analyzed using immunohistochemistry (IHC) for p-ERK, p-AKT, Ki67, and P16. High expression of p-ERK and p-AKT in all GCMN tissues indicates overactivation of the MAPK and PI3K-AKT pathways associated with cell proliferation (Fig. 1c, e). However, Ki67 expression in GCMN tissues was low (average positive rate <5%). In contrast, P16 was extensively expressed (positive rate of 70–90%), indicating its role in GCMN cell cycle arrest (Fig. 1c, e). P16 expression in *NRAS*-mutated GCMN (*n* = 36) was positively associated with the age of the patient (Supplementary Fig. 1g), aligned with the result of the previous study on *Nras*-mutated GCMN mice.^17^ These findings suggest that GCMN cells are predominantly in a senescent-like state that resembles oncogene-induced senescence following RAS/RAF mutation.

Further validation through culturing GCMN cells and immunostaining showed that over 90% of GCMN cells expressed senescence-associated secretory phenotype (SASP) markers (IL-6, SPP1, CCL2, and CXCL8) and over 90% displayed senescence-associated heterochromatin foci (SAHF) marked by co-localization of H3K9me2 and macroH2A (Fig. 1d, f). Moreover, P16 was widely expressed, and Ki67 was expressed at low levels in patient derived GCMN cells, exhibiting senescence characteristics absent in normal melanocytes (Supplementary Fig. 1a, b). Additionally, loss of Lamin B, a senescence biomarker, was observed in GCMN cells (Supplementary Fig. 1c, d). Flow cytometry revealed that approximately 80% of GCMN cells were arrested at the G0/G1 phase, significantly more than normal melanocytes (Supplementary Fig. 1e, f). These findings suggest that GCMN cells exhibit extensive senescence, explaining the limited efficacy of MEK inhibitors in eliminating GCMN.^13,18,19^ Therefore, identifying targets coexpressed in both senescent and proliferative GCMN cells may facilitate complete regression of GCMN.

RNA-seq was performed on GCMN tissues and adjacent normal skin tissues to screen for therapeutic targets. The apoptotic pathway was the most significantly enriched downregulated GO term identified between the GCMN tissues and adjacent normal skin tissues (Supplementary Fig. 1h). Among the apoptotic and antiapoptotic genes, BCL2 exhibited the most pronounced upregulation (Fig. 1g), suggesting its critical role in mediating antiapoptotic activity in GCMN. IHC staining revealed strong and extensive BCL2 expression in GCMN tissues from 50 patients with different gene mutations (positive rate of 80–100%) (Fig. 1h, Supplementary Fig. 1i), which was detected in GCMN cells in both the superficial and deep dermal layers of the skin. Flow cytometry further confirmed high BCL2 expression in more than 98% of GCMN cells compared with only 1% of normal melanocytes, supporting the specificity of BCL2 overexpression in GCMN cells (Supplementary Fig. 1j, k). Co-localization of MelanA and BCL2 was observed by immunofluorescence of GCMN tissues and cells (Fig. 1i–k). Importantly, double immunostaining of P16/BCL2 and Ki67/BCL2 revealed that both senescent and proliferative GCMN cells expressed BCL2 (Fig. 1i–k). These results indicate that the BCL2 expression is a common feature in GCMN cells and may be responsible for the lifelong presence of GCMN cells in the skin observed clinically. Notably, the overexpression of P16 usually triggers cell death^24^; however, the coexpression of BCL2 may prevent GCMN cell apoptosis, playing a crucial role in their sustained survival.

### GCMN mouse models and cellular phenotypes

Next, we aim to establish GCMN mouse models replicated the characteristics and cellular phenotypes observed in human GCMN. Whole-exome sequencing and Sanger sequencing of 50 Asian GCMN patients revealed the predominance of *NRAS*^Q61K^ (42%) mutations (Fig. 1b, Table 1). We generated *Nras*^Q61K^ mutant mice, using Hipp11 (H11) locus knock-in technology (Supplementary Fig. 2a–c), which exhibited darker hair and hyperpigmentation (Fig. 1l), deep dermal infiltration of nevocytes, hypertrichosis, and satellite nevi, mirroring human GCMN (Fig. 1m). Remarkably, the distribution of GCMN lesions in mice aligned with the 6B rule observed in human GCMN, demonstrating bolero, back, bathing trunk, breast/belly, body extremity, and body distribution patterns (Supplementary Fig. 3a). Histological examination revealed abundant melanin and scattered nevocytes (without nest formation) in the skin, tail, paw, and hair follicles (Supplementary Fig. 3b, c).

**Table 1:** Clinical characteristics and genotype of 50 GCMN patients

Patient number	Sex	Age at biopsy date	Mutation type	PAS (cm)	Site or 6B pattern	Krengel classification of main lesion	Satellites	Complications
1	F	8	NRAS Q61K	40–60	Breast/Belly	G1 C0 R2 N0 H0 S2	<20	No
2	M	5	NRAS Q61K	40–60	Body extremity	G1 C0 R1 N0 H2 S1	<20	No
3	F	7	NRAS Q61K	40–60	Bathing trunk	G1 C1 R1 N0 H1 S2	20–50	No
4	F	6	NRAS Q61K	40–60	Back	G1 C0 R1 N0 H2 S3	50–100	No
5	M	5	NRAS Q61K	30–40	Scalp	L2 C0 R1 N2 H0 S0	No	No
6	M	3	NRAS Q61K	40–60	Body extremity	G1 C1 R1 N0 H0 S2	20–50	No
7	M	5	NRAS Q61K	40–60	Back/Breast	G1 C0 R2 N0 H1 S0	No	No
8	F	27	NRAS Q61K	30–40	Scalp/Face	L2 C0 R2 N2 H0 S3	50–100	No
9	M	10	NRAS Q61K	30–40	Scalp/Face	L2 C0 R1 N0 H0 S2	20–50	No
10	F	8	NRAS Q61K	40–60	Back	G1 C0 R1 N0 H0 S2	20–50	No
11	M	4	NRAS Q61K	>60	Body	G2 C0 R2 N1 H1 S3	50–100	Melanoma Development
12	M	22	NRAS Q61K	30–40	Scalp/Face	L2 C1 R1 N0 H0 S0	No	No
13	F	8	NRAS Q61K	40–60	Back	G1 C0 R1 N0 H0 S3	50–100	No
14	M	7	NRAS Q61K	40–60	Back	G1 C2 R1 N0 H0 S1	<20	No
15	M	8	NRAS Q61K	40–60	Body extremity	G1 C0 R1 N0 H2 S0	No	No
16	M	3	NRAS Q61K	40–60	Body extremity	G1 C2 R0 N0 H0 S1	<20	No
17	F	2.6	NRAS Q61K	>60	Bathing trunk	G2 C0 R0 N0 H0 S3	100–200	No
18	F	28	NRAS Q61K	40–60	Bathing trunk	G1 C1 R1 N1 H0 S2	20–50	No
19	M	12	NRAS Q61K	40–60	Bathing trunk	G1 C1 R1 N1 H1 S0	No	No
20	F	3	NRAS Q61K	40–60	Bolero	G1 C1 R0 N0 H2 S1	<20	No
21	M	10	NRAS Q61K	30–40	Scalp	L2 C0 R2 N0 H0 S2	20–50	No
22	M	4	NRAS Q61R	>60	Bolero	G2 C0 R2 N1 H0 S3	>200	No
23	M	7	NRAS Q61R	>60	Bolero	G2 C0 R1 N0 H1 S3	100–200	No
24	M	20	NRAS Q61R	40–60	Breast	G1 C0 R1 N0 H2 S2	20–50	No
25	F	2.4	NRAS Q61R	30–40	Back	L2 C0 R1 N0 H1 S2	20–50	No
26	F	2.6	NRAS Q61R	30–40	Back	L2 C0 R1 N0 H0 S0	No	No
27	M	4	NRAS Q61R	40–60	Bathing trunk	G1 C1 R1 N2 H1 S2	20–50	No
28	M	7	NRAS Q61R	>60	Body	G2 C0 R1 N2 H1 S2	20–50	Melanoma Development
29	F	1.1	NRAS Q61R	30–40	Belly	L2 C2 R0 N0 H0 S0	No	No
30	F	18	NRAS Q61R	20–30	Upper leg	L1 C0 R1 N2 H2 S2	20–50	No
31	F	13	NRAS Q61R	30–40	Upper/Lower arm	L2 C0 R1 N2 H2 S0	No	No
32	F	2.8	NRAS Q61R	40–60	Body extremity	G1 C0 R1 N2 H1 S0	No	No
33	F	7	NRAS Q61R	20–30	Lower arm	L1 C0 R1 N2 H2 S3	50–100	No
34	F	4	NRAS Q61R	30–40	Back	L2 C0 R1 N2 H0 S0	No	No
35	M	4	NRAS Q61R	30–40	Back	L2 C0 R2 N0 H0 S2	20–50	No
36	F	6	NRAS Q61R	>60	Body extremity	G2 C0 R1 N2 H1 S3	50–100	No
37	M	4	BRAF V600E	40–60	Back	G1 C0 R1 N2 H1 S1	<20	No
38	M	12	BRAF V600E	40–60	Bolero	G1 C0 R2 N1 H2 S0	No	Overgrowth lesion
39	F	11	BRAF V600E	40–60	Body extremity	G1 C1 R1 N1 H1 S0	No	No
40	M	4	BRAF V600E	>60	Bolero	G2 C0 R2 N1 H0 S3	>200	Overgrowth lesion
41	F	5	BRAF V600E	>60	Body extremity	G2 C0 R1 N0 H1 S0	No	No
42	F	6	BRAF V600E	40–60	Bathing trunk	G1 C0 R2 N0 H1 S3	50–100	Overgrowth lesion
43	F	9	Wild-type	30–40	Upper/Lower arm	L2 C0 R1 N0 H1 S1	<20	No
44	M	4	Wild-type	40–60	Body extremity	G1 C1 R2 N0 H1 S0	No	No
45	M	6	Wild-type	40–60	Body extremity	G1 C0 R1 N0 H1 S0	No	No
46	F	5	Wild-type	>60	Bolero	G2 C1 R1 N2 H0 S3	100–200	Overgrowth lesion
47	F	5	Wild-type	40–60	Scalp/Face	G1 C0 R2 N1 H2 S0	No	No
48	M	10	ALK Fusion	30–40	Scalp/Face	L2 C0 R1 N0 H0 S1	<20	No
49	F	6	BRAF Fusion	40–60	Bolero	G1 C0 R1 N0 H2 S2	20–50	No
50	M	7	RAF1 Fusion	>60	Bolero	L2 C1 R1 N0 H1 S1	<20	Neurofibroma

These nevocytes expressed human GCMN biomarkers (S100B, TRP1, MITF, HMB45) and stem cell markers highly expressed in human GCMN (Nestin, c-kit, Fascin)^29^ (Supplementary Fig. 1l). High levels of P16, SASP (IL6/CCL2), and BCL2 were also detected (Fig. 1n, Supplementary Fig 1l), indicating that the mouse model replicates human GCMN. The results highlight the roles of P16 and BCL2 in maintaining GCMN. A similar H11-*Tyr*-*Braf*^V600E^ GCMN mouse model (Supplementary Fig. 2d, e) also exhibited phenotypic similarities to human GCMN (Supplementary Fig. 4a). Given the extensive expression of the antiapoptotic protein BCL2 observed in GCMN cells and its ability to prevent P16-induced cell death, BCL2 was identified as a therapeutic target for GCMN.

### Efficacy of BCL2 inhibitors on GCMN cells

**Fig. 2:**
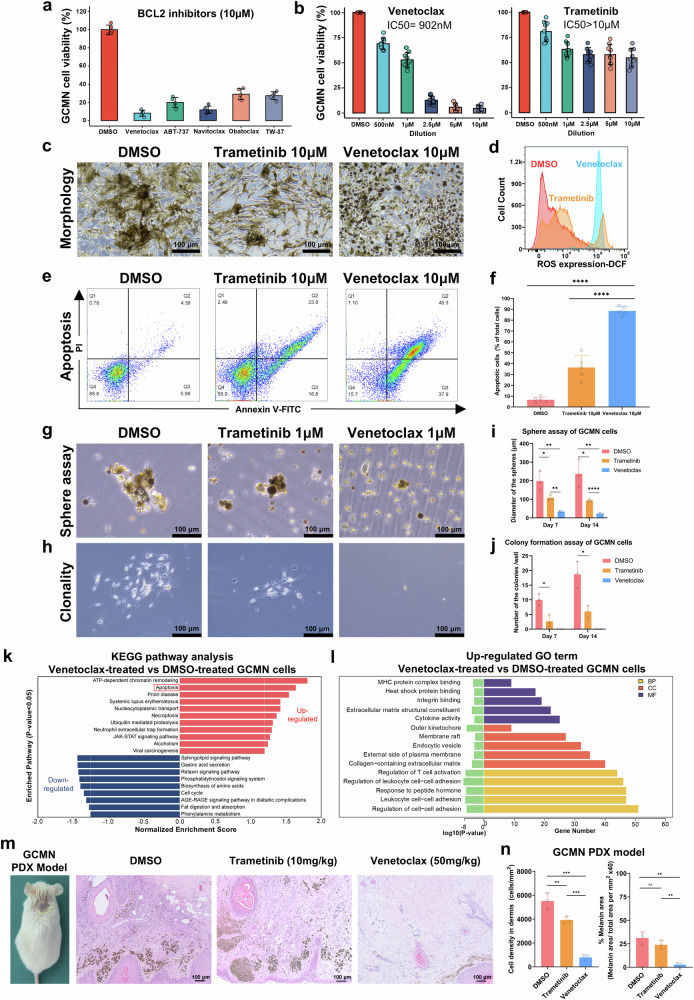
BCL2 inhibitor significantly suppresses sphere and colony formation of human GCMN and eliminates GCMN cells. **a** CCK8 assay evaluating the efficacy of five BCL2 inhibitors in treating GCMN cells from five different patients. Cell viability was assessed after 72 h of inhibitor treatment with 10 μM dilution (*n* = 5). Mean ± SD. **b** CCK8 assay comparing the efficacy of Venetoclax (left graph) and Trametinib (right graph) in treating GCMN cells from nine different patients. Cell viability was assessed after 72 h of inhibitor treatment, with concentration gradients used to evaluate average IC50 (*n* = 9). Mean ± SD. **c** Morphological changes in GCMN cells following 72 h of treatment with DMSO, Trametinib (10 μM), and Venetoclax (10 μM). Scale bar=100μm. **d** Flow cytometry analysis detecting ROS expression in GCMN cells after 24 h of treatment with DMSO, Trametinib (10 μM), and Venetoclax (10 μM). **e** Flow cytometry analysis of apoptotic activity in GCMN cells after 72 h of treatment with DMSO, Trametinib (10 μM), and Venetoclax (10 μM). **f** Percentage of apoptotic cells in GCMN following 72 h of treatment with DMSO, Trametinib, and Venetoclax (*n* = 5). Mean ± SD. **g** Cell sphere assay conducted on GCMN cells after 14 days of low-dose (1 μM). Trametinib and Venetoclax treatment. Scale bar=100μm. **h** Colony formation assay of GCMN cells after 14 days of treatment with low-dose (1 μM) Trametinib and Venetoclax. Scale bar=100μm. **i** Average diameters of the formed spheres following 7 and 14 days of the cell sphere assay with DMSO, Trametinib, and Venetoclax (*n* = 3). Mean ± SD. **j** Average number of colonies formed after 7 and 14 days of colony formation assay with DMSO, Trametinib, and Venetoclax treatment (*n* = 3). Mean ± SD. **k** Significantly up-regulated or down-regulated pathways in Venetoclax-treated (10 μM, 72 h) GCMN cells identified by RNA-seq, comparing DMSO treatment. The apoptosis pathway is labeled with red frame. **l** Significantly up-regulated gene ontology terms in Venetoclax-treated (10 μM, 72 h) GCMN cells identified by RNA-seq, comparing DMSO treatment. **m** Hematoxylin and eosin (HE) staining demonstrating the efficacy of intralesional injection of Venetoclax (50 mg/kg daily for 7 days) and Trametinib (10 mg/kg daily for 7 days) in the constructed GCMN patient-derived xenograft (PDX) model. Scale bar=100μm. **n** Count of the cell density and area of melanin in the dermis based on the HE staining after DMSO, Venetoclax, and Trametinib treatment in GCMN PDX model (*n* = 3). Mean ± SD

Cytotoxicity testing of five leading BCL2 inhibitors (venetoclax, ABT737, navitoclax, obatoclax, and TW-37) on primary GCMN cells identified venetoclax as the most effective (Fig. 2a), with a significantly lower IC50 (902 nM vs. >10 μM) (Fig. 2b, c) compared to trametinib, a MEK inhibitor extensively studied preclinically and clinically. Additionally, high levels of reactive oxygen species (ROS) were detected in venetoclax-treated GCMN cells (Fig. 2d). Flow cytometry analysis revealed that venetoclax induced apoptosis in more than 85% of GCMN cells, which was significantly greater than the percentage of apoptotic cells induced by trametinib (Fig. 2e, f). Additionally, venetoclax demonstrated potent cytotoxicity against GCMN cells from patients with various gene mutations, whereas trametinib was primarily effective against cells with gene fusion (Supplementary Fig. 5a). Venetoclax demonstrated lower cytotoxicity (IC50 > 10 μM) and induced less apoptosis against normal skin fibroblasts, melanocytes, and keratinocytes than GCMN cells (Supplementary Fig. 5b-e). Additionally, venetoclax extensively killed GCMN cells but spared keratinocytes and fibroblasts in the co-culture models (Supplementary Fig. 5f). These findings indicated the specificity of venetoclax for targeting GCMN cells. Given that GCMN cells arise from mutations in neural crest stem cells and melanocyte precursors from mesoderm during embryonic development,^30^ they retain certain stemness traits.^29^ Venetoclax suppressed these GCMN cell stemness traits, reducing sphere (Fig. 2g, i) and colony formation (Fig. 2h, j), which could prove to be crucial for future GCMN treatment and recurrence prevention. RNA-seq was conducted on GCMN cells treated with venetoclax (10 μM for 72 h) and DMSO to investigate the potential mechanisms underlying venetoclax’s cytotoxic effects. Gene set enrichment analysis (GSEA) of differentially expressed genes revealed that venetoclax-treated group upregulated the apoptosis pathway (Fig. 2k) and GO terms involved in immune response, including cytokine activity, MHC protein complex binding, and immune cell activation (Fig. 2l). These findings suggest that venetoclax induces immune-dependent cytotoxic effects. Therefore, investigating the immune response following venetoclax treatment in vivo was essential.

### BCL2 inhibitors effectively target GCMN cells in GCMN patient-derived xenograft (PDX) model mice

To determine whether venetoclax can induce cytotoxicity in human GCMN tissue, we established a patient-derived xenograft (PDX) model in C-NKG mice depleted of T, B, and natural killer (NK) cells and with dysfunctional macrophages. Two months after the engraftment of GCMN tissue, we administered daily intralesional injections of DMSO, trametinib, and venetoclax for 14 days. In our patient-derived xenograft (PDX) model, venetoclax significantly reduced nevus cell density and pigmentation (Fig. 2m, n), confirmed by HE staining and immunostaining (Supplementary Fig. 5h). Ex vivo explant cultures showed disintegration of nevus cell nests, nuclear pyknosis, cellular fragmentation, and reduced S100B+ cells in the dermis following venetoclax treatment (Supplementary Fig. 5g), whereas the DMSO and trametinib groups presented intact nevus cell nests, typical GCMN cellular morphology, and a large proportion of S100B+ cells. BCL2 siRNA silencing in GCMN cells also reduced cell viability and induced apoptosis (Supplementary Fig. 5i–k). Subsequently, cell-derived xenograft models were constructed in which GCMN cells treated with siBCL2 failed to form melanocytic lesions in immunodeficient mice, in contrast to those treated with siNC (Supplementary Fig. 5l). Collectively these findings demonstrate the efficacy of venetoclax in treating GCMN in PDX models and ex vivo models and underscore the potential of targeting BCL2 for GCMN treatment.

### Venetoclax-induced regression in GCMN model mice

**Fig. 3:**
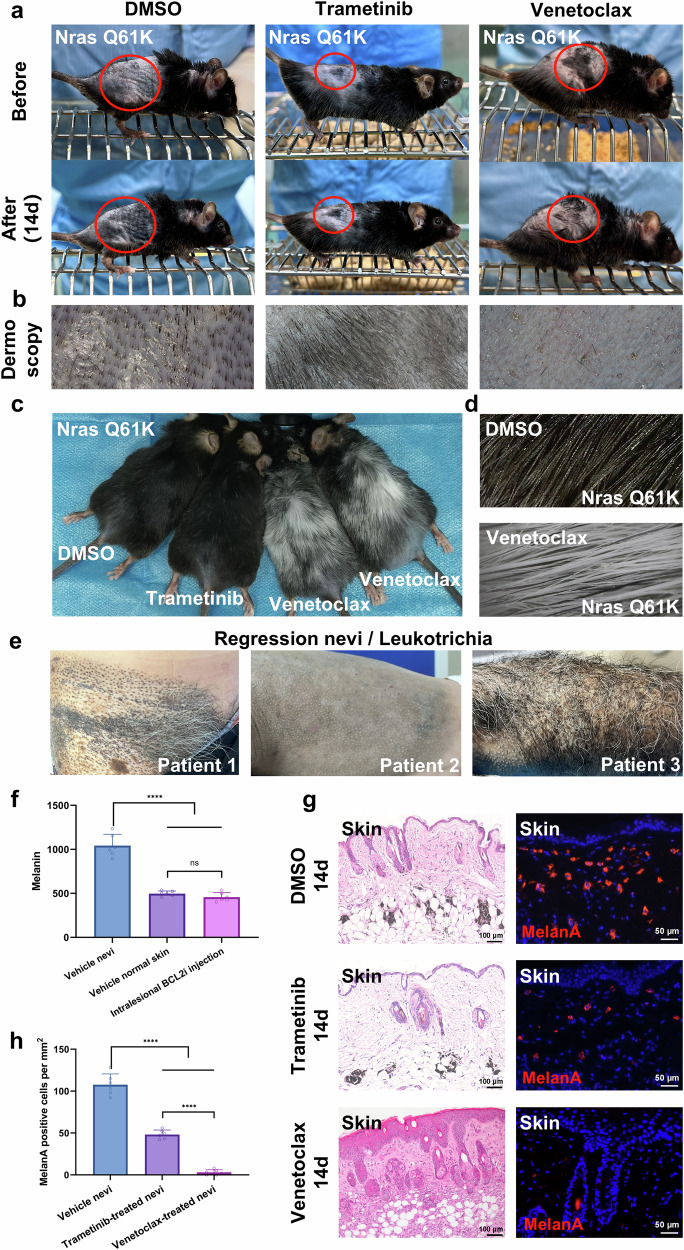
BCL2 inhibitor induces significant regression and leukotrichia in GCMN lesions of H11-*Tyr*-*Nras*^Q61K^ mice. **a**, **b** Assessment of treatment efficacy based on phenotypic and dermoscopic evaluations 14 days post-injection of DMSO, Trametinib, or Venetoclax in the GCMN lesions of H11-*Tyr*-*Nras*^Q61K^ mice. **c**, **d** Phenotypic observations and dermoscopic images of hair regrowth 90 days after intralesional injections with DMSO, Trametinib, or Venetoclax in shaved H11-*Tyr*-*Nras*^Q61K^ mice. **e** Spontaneously regressed human GCMNs exhibiting hypopigmentation and leukotrichia, resembling the regressed mouse GCMN phenotypes. **f** Analysis of melanin content using a Mexameter MX18 detector from the CKMPA10 instrument, performed 14 days after Venetoclax intralesional injection (*n* = 6 mouse for each group). Mean ± SD. **g** Hematoxylin and eosin (HE) staining and immunostaining demonstrating the histological changes and the MelanA+ GCMN cells in the skin after 14 days of intralesional injection with DMSO, Trametinib, or Venetoclax. Scale bar=100μm and 50μm. **h** Bar graph indicating the number of remaining MelanA+ GCMN cells in the skin 14 days after intralesional injection of DMSO, Trametinib, or Venetoclax (*n* = 6 mouse for each group). Mean ± SD

Subsequently, we examined the efficacy of BCL2 inhibitor in GCMN transgenic mice. Since both GCMN patients and GCMN model mice exhibit dark and thick hair (Fig. 1l), assessing changes in hair following drug intervention is crucial. We intralesionally injected DMSO, trametinib, and venetoclax into the back skin and tail of unshaved mice. After 14 days of daily injections, gray hair appeared at the venetoclax injection sites (Supplementary Fig. 6a–c), whereas no such changes were observed in the DMSO and trametinib groups. Histological analysis showed reductions of melanin and nevocytes in tail and hair follicles (Supplementary Fig. 6d). To further investigate the potential of venetoclax to regress nevus lesions, we shaved a group of mice and administered intralesional injections of DMSO, trametinib, or venetoclax directly into the GCMN lesions. After 14 days of daily injection, complete regression of nevus lesions was evident only in the venetoclax-treated group, in contrast with persistent lesions in the DMSO and trametinib groups (Fig. 3a, b).

Remarkably, at 90 days posttreatment, Venetoclax-treated mice presented extensive gray hair (leukotrichia) on their backs (Fig. 3c, d), a phenomenon that was absent in the DMSO- and trametinib-treated mice. Notably, no leukotrichia occurred in wild-type (WT) mice treated with venetoclax (Supplementary Fig. 6e). We analyzed MelanA+ melanocytes in 30 hair follicles from six different mice, comparing WT and GCMN mice following treatment with either Venetoclax or DMSO. Our findings revealed a significant reduction in MelanA+ cells in Venetoclax-treated GCMN mice, with cell numbers lower than those observed in DMSO-treated GCMN mice and Venetoclax-treated WT mice (Supplementary Fig. 6f, g). MelanA+ cells persisted in hair follicles of WT mice after Venetoclax treatment. These results are consistent with the phenotypic differences observed between GCMN and WT mice. Clinical observations of regressed GCMN commonly include faded lesions accompanied by extensive leukotrichia (Fig. 3e), and these features were also present in the venetoclax-treated GCMN mice in this study. Histological analysis post-venetoclax treatment revealed clearance of nevus cells and melanin (Fig. 3g, h), along with dermal infiltration of immune cells (Fig. 3g). The melanin content markedly decreased after treatment, approaching levels characteristic of normal mouse skin (Fig. 3f).

Similarly, intralesional injection experiments on H11-*Tyr*-*Braf*^V600E^ mutant GCMN model mice confirmed venetoclax-induced nevi lesion regression and leukotrichia, which contrasted with the outcomes of the DMSO treatments (Supplementary Fig. 4b, c). Oral administration of venetoclax over six weeks also induced hypopigmentation and leukotrichia in GCMN lesions (Supplementary Fig. 7a, b), accompanied by nevus cell clearance and reduced melanin content (Supplementary Fig. 7c, d). Moreover, no apparent organ toxicity and hematological abnormalities were observed following venetoclax treatment (Supplementary Fig. 7g, h). Notably, after 335 days of follow-up, no GCMN recurrence occurred in the venetoclax-treated mice (Supplementary Fig. 7e). Instead, numerous resident immune cells were found in the dermis, indicating a persistent immune response (Supplementary Fig. 7e).

**Fig. 4:**
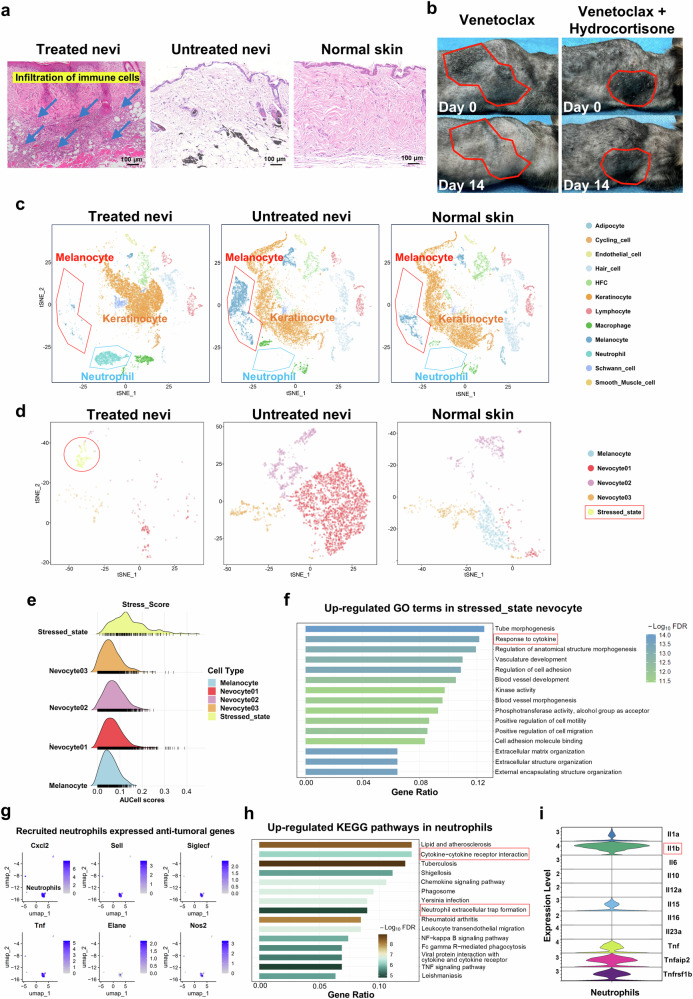
Single-cell sequencing reveals neutrophil-mediated immune response in Venetoclax-treated GCMN lesions. **a** HE staining displaying histological features of Venetoclax-treated nevi, untreated nevi, and normal skin from the mouse. Scale bar=100μm. **b** Phenotypes of mouse GCMN lesions showing reduced efficacy in the group with simultaneous injection of Venetoclax and Hydrocortisone. **c** t-SNE visualization illustrating cell clusters for each group separately, including Venetoclax-treated GCMN, untreated GCMN, and normal skin. Single-cell sequencing was conducted three days after intralesional injection. **d** t-SNE visualization depicting melanocyte subclusters among the three groups, presented separately. **e** Stress response scores of all melanocyte subclusters. **f** Up-regulated gene ontology terms in stressed-state nevocytes. **g** Expression of anti-tumoral genes in recruited neutrophils in BCL2i-treated GCMN. **h** Up-regulated KEGG pathways in recruited neutrophils in BCL2i-treated GCMN. **i** Violin plots displaying the expression of cytokines in the neutrophil cluster

The infiltration of immune cells underscore the potential role of the immune response in increasing the efficacy of BCL2i treatment (Fig. 4a). Additionally, the plasma concentration of Venetoclax peaked at 1.75 μg/ml after 8 h of intralesional injection (Supplementary Fig. 8b), which is lower than the concentration (5-10 μg/ml) required to completely eliminate mouse GCMN cells in vitro (Supplementary Fig. 8a). This suggests a possible involvement of the immune response. Furthermore, we administered venetoclax in combination with hydrocortisone intralesionally to GCMN model mice (Fig. 4b), resulting in reduced hypopigmentation and highlighting the critical importance of immune responses. To elucidate the mechanism by which the immune response orchestrates the elimination of GCMN lesions, single-cell sequencing was subsequently conducted.

### Neutrophil-Mediated Immune Response in Regressed GCMN Lesions

Single-cell sequencing on samples from venetoclax-treated nevi (14 d) revealed that venetoclax-treated nevi exhibited a significant presence of neutrophils (Fig. 4c, Supplementary Fig. 8c–e) and a significant reduction in the nevocyte (Fig. 4d, Supplementary Fig. 8f, g), suggesting a critical role for neutrophils in effective immune activation against nevocytes by upon BCL2 inhibitor (venetoclax) treatment. Within the melanocyte clusters, we observed a reduction in most nevocyte subclusters after BCL2 inhibitor treatment, accompanied by the emergence of a new nevocyte subcluster characterized by high oxidative stress (designated the “stress_state”) (Fig. 4d, e). These nevocytes expressed the apoptotic gene CASP3 (Supplementary Fig. 8h), indicating ongoing apoptosis. GO enrichment analysis revealed significant enrichment of pathways involved in the interleukin response in the upregulated genes of this cluster (Fig. 4f), suggesting a role for interleukins in inducing nevocyte apoptosis. Neutrophils were absent in untreated nevi and normal skin but were abundant in venetoclax-treated nevi, where they expressed biomarkers characteristic of activated neutrophils (Fig. 4g). The upregulation of cytokine, interleukin, and NET pathways in neutrophils supports their involvement in nevocyte elimination via NETosis (Fig. 4h).

Our further analysis revealed that the Il1b-Il1r1 ligand-receptor interaction was crucial for communication between neutrophils and apoptotic nevocytes. Il1b was the most highly expressed interleukin among the top 20 genes overall in neutrophils (Fig. 4i, Supplementary Fig. 8i), underscoring its pivotal role in the neutrophil-mediated immune response. Il1b is known to activate NETosis in neutrophils, emphasizing its importance in this context. Moreover, cell communication analysis revealed increased interactions in Venetoclax- treated nevi, with specific involvement of the Il1 pathway (Supplementary Fig. 8j, k). In particular, communication via the Il1 pathway was observed between neutrophils and apoptotic nevocytes (Supplementary Fig. 8l), facilitated by high expression of Il1 receptors across all nevocyte clusters (Supplementary Fig. 8m). Notably, the Il1b-Il1r1 ligand‒receptor interaction contributed significantly to this communication (Supplementary Fig. 8n), highlighting the importance of Il1b in mediating interactions between neutrophils and apoptotic nevocytes.

In summary, following BCL2 inhibitor injection, neutrophils are recruited to GCMN lesions where they induce NETosis to eliminate nevocytes via the Il1b pathway, ultimately driving the regression of GCMN lesions. Additionally, a cluster of proliferative and reparative keratinocytes expressing Krt6a and Krt16 appeared post-venetoclax injection (Supplementary Fig. 9a–d). These keratinocytes presented increased cell bridging, increased collagen production (Supplementary Fig. 9e), and increased cell proliferation pathways (Supplementary Fig. 9f), suggesting a self-healing response of the skin to inflammation following BCL2 inhibitor treatment. These findings indicate that neutrophils are recruited to GCMN lesions.

### Validation of neutrophil involvement in nevus regression

**Fig. 5:**
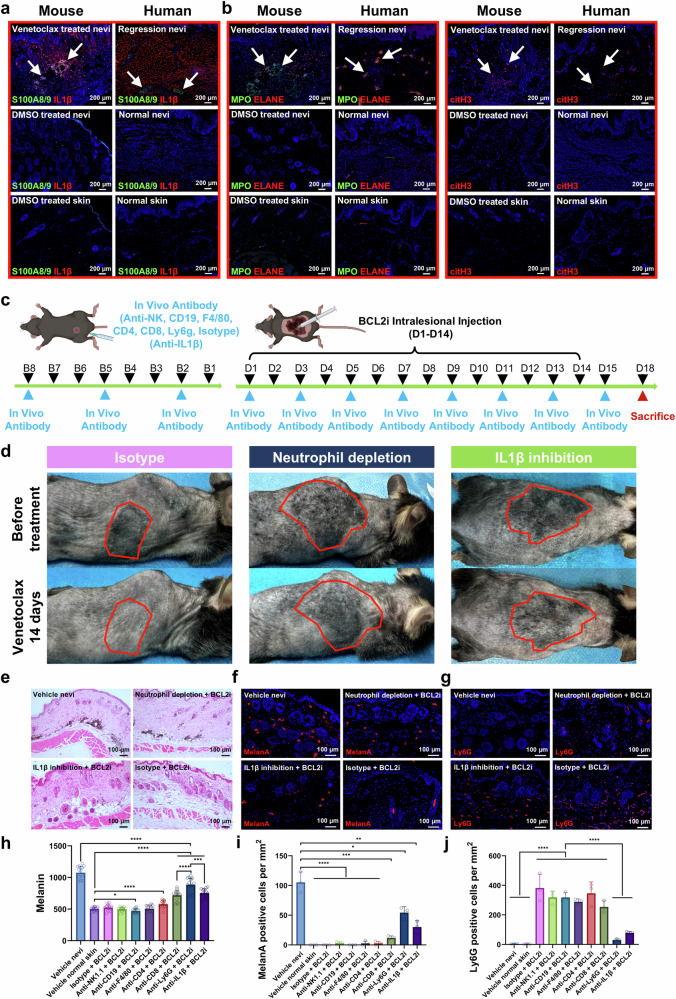
Depletion of neutrophils and IL1β inhibition diminishes the efficacy of Venetoclax in treating GCMN. **a** Immunostaining detecting S100A8/9 + IL1β+ neutrophil infiltration in regressed nevi, unregressed/untreated nevi, and normal skin of mouse and human. Scale bar=200μm. **b** Immunostaining identifying neutrophil extracellular trap formation (MPO + , ELANE + , and citH3 + ) in regressed nevi, unregressed/untreated nevi, and normal skin of mouse and human. Scale bar=200μm. **c** Diagram illustrating the timeline of in vivo cell depletion accompanied by intralesional BCL2i injection in *Nras*-mutated GCMN mice. **d** Phenotypes of GCMN lesions showing the reduced efficacy of Venetoclax treatment in groups with neutrophil depletion and IL1β inhibition. The red frames indicate the area of Venetoclax-injected GCMN lesions. **e** HE staining demonstrating retained nevocytes and melanin in GCMN lesions in the group with neutrophil depletion and IL1β inhibition. Scale bar=100μm. **f**, **g** Immunostaining showing retained MelanA+ nevocytes and reduced Ly6G+ neutrophils in GCMN lesions in the group with neutrophil depletion and IL1β inhibition. Scale bar=100μm. **h** Melanin content analysis of GCMN lesions undergoing BCL2i treatment in groups with different antibody injections (*n* = 15, 3 mouse with 5 different locations for each group). Mean ± SD. **i**, **j** The positive MelanA+ nevocytes and Ly6G+ neutrophils per mm^2^ in groups with different antibody injections (*n* = 3 mouse for each group). Mean ± SD

Immunostaining confirmed the presence of IL1β+ neutrophils in venetoclax-treated nevi (Fig. 5a), aligning with scRNA-seq results. Further staining for NET biomarkers, including MPO, ELANE, and Citrullinated Histone H3, confirmed their presence and suggested the formation of NETs by these neutrophils (Fig. 5b). Similar findings were observed in spontaneously regressed human GCMN lesions, which revealed comparable neutrophil infiltration, IL1β expression (Fig. 5a), and NETs formation (Fig. 5b) in regressed nevi but not in unregressed nevi or normal skin.

We then injected antibodies into H11-*Tyr*-*Nras*^Q61K^ GCMN mice to selectively deplete various immune cell lineages. This included injections to deplete CD4 + T cells, CD8 + T cells, B cells, macrophages, NK cells, and neutrophils, and an isotype control was also implemented in this experiment with confirmed successful depletion of each immune cell lineage (Supplementary Fig. 10a). The efficacy of venetoclax treatment in nevus regression was assessed over a 14-day period after intralesional injection (Fig. 5c). Remarkably, compared with the isotype control, depletion of neutrophil but not other immune cells resulted in most significant reduction in the efficacy of venetoclax (Fig. 5d, Supplementary Fig. 10c). T-cell depletion only slightly reduced the efficacy of venetoclax. Analysis of melanin content (Fig. 5e, h) and Melan-A+ nevocytes (Fig. 5f, i) revealed significantly greater melanin content and approximately 52% of Melan-A+ nevocytes surviving in the neutrophil-depleted group than in the other depletion groups. Significant neutrophil infiltration was observed in all of the other groups except for the neutrophil- depleted group (Fig. 5g, j).

Long-term follow-up immunostaining of the regressed lesions revealed that the resident immune cells were primarily neutrophils (Supplementary Fig. 7f), with minor populations of T cells and B cells, further emphasizing the importance of neutrophil-mediated immune responses. This phenomenon was also observed in patients with spontaneous regression of GCMN, highlighting the conserved mechanism of IL1β+ neutrophil-mediated NETosis in GCMN lesion regression (Fig. 5a, b). Additionally, epidermal thickening and KRT6A+ keratinocytes were specifically observed in both venetoclax-induced regressed mouse GCMN and human regressed GCMN lesions (Supplementary Fig. 9g, h) and were absent in DMSO-treated/unregressed GCMN lesions, venetoclax-treated normal skin, and DMSO-treated normal skin. These results suggest that the alternation of keratinocytes was associated with the presence of GCMN cells treated with venetoclax.

Pretreatment with IL1β specific inhibition (Supplementary Fig. 10b) resulted in a slight but significant reduction in the efficacy of venetoclax (Fig. 5d). Melanin contents remained elevated compared with those in normal skin (Fig. 5e, h), and Melan-A+ nevocytes were still present in the dermis (Fig. 5f, i). Neutrophil infiltration was significantly lower in the IL1β inhibition group than in the isotype control group (Fig. 5g, j). Furthermore, reduced leukotrichia were observed in both the neutrophil depletion and the IL1β inhibition groups (Supplementary Fig. 10d).

**Fig. 6:**
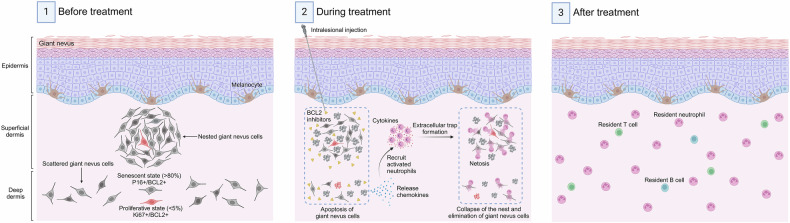
Diagram illustrating the total elimination of giant nevus cells achieved by anti-BCL2 therapy via senolytic and immune induction. This figure was created with BioRender (https://www.biorender.com/) and is permitted for publication

The findings collectively highlight the potential of targeting BCL2 for GCMN treatment. Venetoclax effectively induces apoptosis in GCMN cells, with a critical role for neutrophils and the IL1β pathway in mediating immune responses and nevus regression. These results provide a basis for further clinical investigation of BCL2 inhibitors in GCMN therapy (Fig. 6).

## Discussion

GCMNs have lifelong life-threatening risks of developing melanoma^3–5^ and neurocutaneous melanocytosis.^6,7^ Effective therapies capable of completely eliminating GCMN are lacking in clinical practice. Our study reveals that GCMN cells exhibit a cellular phenotype of growth arrest and prolonged survival, as indicated by the extensive coexpression of P16 and BCL2. Various senescent cell characteristics were identified in most GCMN cells through immunostaining, suggesting that the senescent state of GCMN cells contributes to the limited efficacy of MEK inhibitors in clinical settings. This highlights the need for alternative therapies targeting both proliferative and senescent nevus cells.

We observed extensive and specific expression of BCL2 in both senescent and proliferative GCMN cells, making BCL2 a promising therapeutic target. BCL2 is an antiapoptotic protein that promotes cell survival by inhibiting apoptosis.^27,31^ BCL2 inhibitors (BCL2i) are designed to reverse these antiapoptotic effects, thereby promoting cell death. These inhibitors have shown efficacy in treating proliferative tumors, such as acute myeloid leukemia,^32^ and diseases associated with cellular senescence, such as diabetes^33^ and Alzheimer’s disease.^34^ We propose that BCL2 inhibitors can eliminate both proliferative and senescent GCMN cells due to their high BCL2 expression.

Using an *Nras*^Q61K^-transgenic GCMN mouse model, which mimics the common mutation in Chinese GCMN patients, we demonstrated that venetoclax, a BCL2 inhibitor, effectively induces hypopigmentation and clears nevus cells via intralesional injection or oral administration. Long-term follow-up revealed no recurrence of skin hyperpigmentation, and similar efficacy was observed in *Braf*^V600E^ transgenic mice. Venetoclax achieved more effective clearance of GCMN cells than MEK inhibitors, highlighting its potential for achieving complete regression of GCMN lesions in clinical settings.

GCMN cells are believed to originate from hair follicle stem cells and other precursors,^29,35^ underscoring the importance of targeting hair follicles to prevent GCMN recurrence. Extensive leukotrichia (gray hair) and reduced melanocytes in hair follicles were developed on GCMN lesions in transgenic mice following venetoclax treatment, suggesting the elimination of nevocytes in hair follicles. Clinically, GCMN regression is rare and typically presents as hypopigmentation and leukotrichia, similar to our model mice following treatment. These effects were not observed in wild-type mice or patients receiving venetoclax for leukemia.^36,37^ However, the product monograph of Venclexta (AbbVie) reported that daily venetoclax administration ( ≥ 6 mg/kg) for three months caused white hair discoloration in dogs. This discrepancy may be attributed to the shorter treatment duration (14 days) and lower drug dosage (50 mg/kg on mice, equivalent 1-2 mg/kg on dog) used in our study. However, low-dose, short-term venetoclax treatment can induce significant cytotoxicity in GCMN cells, suggesting a heightened sensitivity of GCMN to venetoclax. Our cell viability assays also support this observation. This increased sensitivity may be due to the hyperactivation of the PI3K-AKT pathways in GCMN, leading to the upregulation of BCL2^38^ and, consequently, a stronger dependence on BCL2 for cell survival.

Our study also indicates the participation of the immune response during GCMN regression, as evidenced by substantial immune cell infiltration following BCL2i injection. Single-cell sequencing of venetoclax-treated mice revealed the crucial role of the neutrophil-mediated immune response, characterized by increased neutrophil numbers, IL1β expression, NETosis, and enhanced communication between neutrophils and apoptotic GCMN cells. Neutrophil depletion and IL1β suppression impaired the efficacy of BCL2 inhibitors, highlighting the importance of the immune response. Resident neutrophils persisted in the dermis of treated lesions without recurrence during long-term follow-up. These findings suggest that neutrophils play an indispensable role in GCMN regression, exerting cytotoxic effects and enhancing the efficacy of BCL2 inhibitors.

However, how BCL2 inhibitors trigger NETosis was not fully elucidated in this study. The probable mechanism is that the BCL2 inhibitors induced extensive apoptosis of GCMN cells (Fig. 2f), leading to an increase and accumulation of reactive oxygen species (ROS) (Fig. 2d). ROS is an important trigger promoting the formation of NETosis in neutrophils, which may subsequently amplify the cytotoxic effect on GCMN cells.^39,40^ Further research investigating the relationship between anti-BCL2 and NETosis formation would be intriguing. NETs have been reported to play dual roles in tumor progression, acting as both pro-tumorigenic and anti-tumorigenic factors depending on the context.^41^ Previous studies have shown that NETs can promote tumor progression by fostering an inflammatory microenvironment, enhancing cancer cell migration, and shielding tumor cells from immune clearance.^41^ However, our results indicate an anti-tumorigenic role of NETs in the regression of GCMN following BCL2 inhibition. This discrepancy may be attributed to differences in tumor models, microenvironmental factors, and specific triggers of NETosis. In our study, NETosis was induced in a setting of massive apoptosis and ROS accumulation, which could create a cytotoxic environment unfavorable for tumor cell survival. Additionally, previous studies have reported that NETs can exert direct cytotoxic effects on tumor cells through ROS-dependent mechanisms.^42^

BCL2 inhibitors are reported to synergize with NK cells^43^ and increase the number of T effector memory cells, augmenting antitumor activity. The synergistic effect between BCL2i and neutrophils in eliminating pathological cells warrants further investigation. Recent studies have demonstrated the antitumoral effects of neutrophils,^44–46^ showing their accumulation in successfully immunotherapy-treated tumor tissues and reduced efficacy upon neutrophil blockage.^46^ Another breakthrough study revealed that neutrophils are recruited to eliminate remaining melanoma cells after T-cell immunotherapies.^47^ Since our study demonstrated that neutrophils synergize with venetoclax in regressing GCMN, isolating resident neutrophils from venetoclax-treated GCMN lesions for the development of GCMN immunotherapy represents a promising avenue for future research. Fisher et al^17^ applied the partial antigen SADBE to transgenic mice and GCMN PDX model mice, inducing inflammation and recruiting macrophages that significantly cleared GCMN cells. Similarly, this study also underscores the critical role of activating the immune system in eliminating GCMN, highlighting its potential value in future therapeutic approaches.

The limitations of this study include not testing BCL2 inhibitors in GCMN-associated melanoma and not evaluating the development of malignancies in the mouse models. Additionally, the pathology of GCMN mice in this study has not fully replicated human GCMN (without nest formation), which may lead to potential bias. In summary, this study demonstrates the remarkable efficacy of BCL2 inhibitors in treating GCMN in vivo and in vitro, suggesting their translational potential for future clinical trials.

## Materials and methods

### Patients and sample collection

We enrolled 50 patients diagnosed with GCMN at Shanghai Ninth People’s Hospital between 2020 and 2023. The Kregel classification and 6B distribution were utilized to evaluate the phenotypes of each patient (Table 1). Surgical resection of GCMN lesions was performed on all participants. GCMN samples were collected from the darkest part of the main lesions (satellite lesions were not included) followed by whole exome sequencing, Sanger sequencing, or RNA sequencing to identify driver gene alterations. This study was approved by the Institutional Review Board (IRB) of Shanghai Ninth People’s Hospital (Approval No. 201743), and informed consent was obtained from each patient.

### Immunostaining

Formalin-fixed paraffin-embedded sections were deparaffinized in xylene and dehydrated with a series of ethanol solutions of increasing concentration. Antigen retrieval was performed by incubating the sections with citrate buffer (pH = 6) or EDTA buffer (pH = 9) at 98 °C for 30 min. Endogenous peroxidase activity was blocked with 3% H2O2 for 15 min. The sections were subsequently washed with phosphate-buffered saline (PBS) and blocked for 1 h with PBS supplemented with 5% normal goat serum to minimize nonspecific staining. The sections were incubated with different primary antibodies diluted in blocking buffer overnight at 4 °C. The primary antibodies used in this study are detailed in the antibody section.

For immunohistochemistry, the sections were washed with PBS followed by incubation with an HRP-conjugated secondary antibody for 30 min and then visualized using 3-amino-9-ethylcarbazole (AEC) as a chromogen. The slides were subsequently incubated with hematoxylin for 3 min for nuclei counterstaining.

For immunofluorescence, the sections were washed with PBS followed by incubation with the secondary antibodies for 30 min at 20-30°C. The slides were washed with PBS and were subsequently incubated with DAPI for 5 min to stain the nuclei. All antibodies used in this study are listed in Supplementary Materials.

### Cell culture

Nevocytes were isolated from human GCMN tissue. Tissue samples were cut into 2 mm strips and incubated overnight at 4 °C in 0.25% Dispase II (D4693, Sigma‒Aldrich) in serum-free Dulbecco’s modified Eagle’s medium (DMEM) without Ca^2+^ or Mg^2+^. After the initial incubation, the epidermis was manually removed from the dermis. The dermis was digested for 3–5 h at 37 °C in 1 mg/ml collagenase type IV (C4-BIOC, Sigma‒Aldrich). The suspensions were diluted in DMEM and filtered through 100 mm cell strainers. The cells obtained from the dermal components of each lesion were subsequently cultured in complete MelM medium (2201, ScienCell). Normal melanocytes were isolated from human foreskin. The tissues were incubated in 0.25% Dispase II at 4 °C overnight. The epidermis was manually peeled from the dermis and incubated at 37 °C in 0.25% trypsin (25200056, Gibco) for 15 min. The suspensions were filtered through 100 mm cell strainers and cultured in complete MelM media.

### Cell immunofluorescence staining

Cultured cells were rinsed with PBS, fixed in 4% PFA for 10 min, permeabilized with 0.1% Triton-X 100 for 10 min, and blocked in 3% BSA to block nonspecific binding for 1 h. The cells were incubated with different primary antibodies diluted in blocking buffer overnight at 4°C. After washing with PBS, the cells were incubated with Alexa Fluor 488-labeled secondary antibody or Alexa Fluor 647-labeled secondary antibody for 1 h at 20-30°C. The nuclei were counterstained with DAPI.

### Positive rate evaluation of Immunostainings

For immunohistochemistry, HE and MelanA staining were initially performed to locate the GCMN cell. The red staining (AEC positive) within the GCMN cells was assessed, and the positive rate was calculated using ImageJ (version 1.54) at a 100x magnification. For immunofluorescence staining, the co-localization of fluorescence and DAPI was evaluated and quantified using ImageJ at 100x or 200x magnification. The positive rate was determined and averaged by three independent observers.

### Cell viability assay

Cells (1×10^4^) were plated in 96-well plates, and after 24 h, they were treated as indicated in the figures and assayed for viability after 72 h with a CCK8 kit (Beyotime Institute of Biotechnology, Shanghai, China) according to the manufacturer’s instructions. Inhibitors used in this study: Venetoclax (S8048, Selleck), ABT-737 (S1002, Selleck), Navitoclax (S1001, Selleck), Obatoclax (S6709, Selleck), TW-37 (S1121, Selleck), and Trametinib (S2673, Selleck).

### Flow cytometry analysis

For biomarker detection, cells were harvested and suspended in PBS containing 10% FBS (5×10^6^ cells/ml). They were then stained with conjugated primary antibodies for 1 h at 37 °C in the dark. Cell cycle analysis was performed using the Cell Cycle and Apoptosis Analysis Kit (C1052, Beyotime) in accordance with the manufacturer’s instructions. For cell apoptosis detection, samples were prepared with the Annexin V-PE Apoptosis Detection Kit (C1065, Beyotime) following the manufacturer’s guidelines. Reactive oxygen species (ROS) detection was conducted using the ROS Assay Kit (S0033, Beyotime) as per the manufacturer’s instructions. After washing the cells with PBS, the samples were analyzed using a flow cytometer (Agilent NovoCyte). The resulting data were processed using NovoExpress™ software and FlowJo.

### Sphere assay and colony formation assay

For sphere assay, cells (8×10^3^) were plated in 6-well ultra-Low attachment plates (3471, Corning) and cultured in FBS-free MelM medium with or without 1 µM inhibitors. DMSO was used as the control. Nevosphere diameters were measured at 7 days and 14 days of culture. For colony formation assay, one thousand cells were plated on 6 cm plates in FBS-free MelM medium. After 48 h, the medium was supplemented with 1 µM inhibitors. The medium and drugs were replaced every 3 days. The cell colonies of each well were calculated after 7 and 14 days of culture.

### Small interfering RNA transfection

Cells were seeded in a 6-well plate with antibiotic-free culture medium at 50-60% confluence 24 h before transfection. Small interfering RNA (50 nM) was diluted in 100 µL of Opti-MEM medium (31985070, Thermo Fisher). In a separate tube, 5 µL Lipofectamine 3000 Transfection Reagent (L3000015, Invitrogen) was diluted in 100 µL of Opti-MEM. Both two tubes were incubated for 5 min at 20-30°C. The diluted siRNA and the diluted Lipofectamine RNAiMAX were mixed and incubated for 20 min at 20-30°C to form siRNA-lipid complexes. The complexes were added to the cell dish and incubated at 37 °C for 48 h. A non-targeting control was included in every experiment. siBCL2 sequence (5’-3’): GUGAUGAAGUACAUCCAUUAUTT.

### Real-time qPCR

Color Reverse Transcription Kit (A0010CGQ, EZBioscience) was used to synthesize cDNA following the manufacturer’s instructions. SYBR Green qPCR Master Mix (A0001, EZBioscience) was applied for constructing qPCR reacting mixture. The plate was run using QuantStudio 5 Real-Time PCR Systems (A28575, Thermo Fisher) under the following conditions: initial denaturation at 95 °C for 10 min, followed by 40 cycles of 95 °C for 15 s and 60 °C for 1 min. Relative expression of target genes was normalized by GAPDH and was calculated using the ΔΔCt approach.

BCL2Primers used for qPCR:

BCL2-F: TCTTCAGGGACGGGGTGAAC

BCL2-R: GACATCTCCCGGTTGACGCT

### Ex vivo assays

Surgical excision specimens of human GCMN and normal skin were obtained, and the subcutaneous tissue was meticulously removed. The samples were then trimmed into 0.5 × 0.5 cm explants. These explants were subsequently cultured in organotypic culture media, MELM, for 5 days. During this culture period, some explants were treated with drugs, including trametinib (50 μM) or venetoclax (50 μM), whereas others served as untreated controls. Following the 5-day incubation period, the explants were carefully harvested and washed with phosphate-buffered saline to remove any residual culture medium. The samples were subsequently fixed in 4% PFA solution and then embedded in paraffin. HE staining was performed to evaluate the histological changes posttreatment.

### PDX model and drug administration

The animal study protocol of this research was approved by the Animal Care and Use Committee (ACUC) of Shanghai Ninth People’s Hospital (Approval No. SH9H-2023-A891-1). Randomization and blinding methods were not used for animal studies in this research. After surgical excision, full-thickness GCMN tissues of 1 cm^2^ were xenografted on the backs of C-NKG mice aged 6 weeks (3 mouse for each group). Intradermal injections of venetoclax (50 mg/kg, equivalent to 1-2 mg/kg in human) or trametinib (10 mg/kg, equivalent to 0.2-0.4 mg/kg in human) diluted in 5% Tween 80, 10% DMSO, 40% PEG300, and 45% ddH2O were then performed daily on the xenografts for 14 days. Xenografts were harvested 3 days after the end of the injections. The grafts were then fixed in 4% PFA for histologic analysis.

### CDX model construction

A total of 5×10^6^ GCMN cells were suspended in 200 µl MelM medium and subsequently injected subcutaneously into nude mice aged 6 weeks. Two months post-injection, the skin of the injected mice was harvested to assess the formation of melanocytic lesions.

### Transgenic GCMN mouse model

For *Nras*^Q61K^ mutated GCMN mice, CRISPR/Cas9 technology was used to obtain Tyr-LSL-*Nras*^Q61K^ knock-in mice at the H11 locus. The knock-in gene was specifically expressed in melanocyte lineage cells. The F1 generation mice were then crossed with Dppa3-Cre mice to obtain F2 generation mice with sheared LSL fragments (i.e., the target mice). We initially chose to generate *Tyr* promoter-LSL-*Nras*^Q61K^ mice out of concern that directly constructing *Tyr* promoter-*Nras*^Q61K^ mice might result in embryonic lethality. *Dppa3*-Cre was selected because *Dppa3* is a maternal effect gene specifically expressed in primordial germ cells, oocytes, preimplantation embryos, and embryonic stem (ES) cells. This makes *Dppa3*-Cre a useful tool for inducing mutations during early embryonic development. For *Braf*^V600E^ mutated GCMN mice, *Tyr* promoter-*Braf*^V600E^-WPRE-pA cassette knock-in mouse model was generated at H11 gene locus via CRISPR/Cas9 technology without using the LSL strategy. All the animal experiments adhered to the relevant guidelines and regulations.

### Drug treatment protocol for GCMN model mice

Six weight-matched GCMN mouse (*Nras*-mutated or *Braf*-mutated) with 6 weeks of age were included in each group. Venetoclax (50 mg/kg, equivalent to 1-2 mg/kg in human) or trametinib (10 mg/kg, equivalent to 0.2-0.4 mg/kg in human) was diluted in 10% DMSO, 45% PEG300, and 45% ddH2O for intralesional injection. Intralesional injection of the drug was performed daily for 14 days. Oral gavage of venetoclax (100 mg/kg, equivalent to 2-4 mg/kg in human) was performed every 3 days for 42 days. Some of the mice were sacrificed 3 days after treatment for pathological experiments and single-cell sequencing, and the other mice were sacrificed after 335 days of follow-up.

### Melanin quantitative analysis

A skin detector equipped with a Skin Colorimeter (Cutometer Dual Mexameter® MX 18, Courage + Khazaka) was used to quantify the melanin content of the skin of the GCMN model mice. Untreated nevi lesions, normal skin, and nevi lesions treated with a subcutaneous injection of venetoclax were analyzed, and the melanin content was quantified via statistical analysis.

### Single-cell sequencing of mouse tissues

Age- and weight-matched female *Tyr*-*Nras*^Q61K^ mice were paired, with intervention group receiving anti-BCL2 treatment and control group receiving DMSO. After two weeks of intralesional injection into nevus tissue, the mice were euthanized. Samples were collected from the back nevus tissues of the treated mice, back nevus tissues of the untreated mice, and normal skin tissue from the control mice. These samples were subsequently digested using Whole Skin Dissociation Kit (130-101-540, Miltenyi Biotec) at 37°C for 6 h following the manufacturer’s instruction, and were processed on GentalMACS Dissociator (130-093-235, Miltenyi Biotec). The total epidermal and dermal cell suspensions contained more than 80% live cells, with a concentration of 800–1300 cells/µL. The experimental procedures for scRNA-seq followed established techniques using the Chromium Single-cell 3’ Library V2 Kit (10x Genomics). Methods of analyzing scRNA-seq and RNA-seq data in this study are described in Supplementary Materials.

### In vivo depletion of immune cells

On days 8, 5, 2 prior to BCL2 inhibitor treatment, 750 μg of the following antibodies were administered via intraperitoneal injection: anti-mouse/rat IL1β-InVivo (A2142, Selleck), anti-mouse NK1.1-InVivo (A2142, Selleck), mouse IgG2b isotype control-InVivo (A2145, Selleck), anti-mouse CD8α-InVivo (SA2102, Selleck), anti-mouse CD19-InVivo (A2149, Selleck), anti-mouse CD4-InVivo (A2101, Selleck), anti-mouse Ly6G-InVivo (A2158, Selleck), and anti-mouse F4/80-InVivo (A2155, Selleck). Additionally, 300 μg of these antibodies were administered on days 1, 3, 5, 7, 9, and 11, 13, 15 during BCL2 inhibitor treatment.

### Statistical analysis

One-way ANOVA with Tukey’s multiple comparison test was conducted to compare differences among more than two unpaired groups. The Student’s unpaired t test was utilized to analyze differences between two groups. A p-value of less than 0.05 was considered statistically significant. All measurements were taken from distinct samples. Error bars demonstrate the standard deviation.

## Supplementary information

Supplementary Figures and Materials

## Data Availability

The data from scRNA-seq and RNA-seq in this study will be released from the Sequence Read Archive (SRA) after publication, BioProject number PRJNA1242731.
