# Supplementary material for: Anti-BCL2 therapy eliminates giant congenital melanocytic nevus by senolytic and immune induction
Source: Signal Transduct Target Ther. 2025 May 16;10:161. doi: 10.1038/s41392-025-02247-2 (PMC12081681; doi:10.1038/s41392-025-02247-2)
Supplement: Supplementary file 1 — Supplementary Figures and Materials [file 41392_2025_2247_MOESM1_ESM.docx]

Supplementary Materials for

**Anti-BCL2 therapy eliminates giant congenital melanocytic nevus by senolytic and immune induction**

Boxuan Wei^1#^, Qingxiong Yu^1#^, Jiamin Jin^1^, Danli Zhu^1^, Bohan Lai^1^, Jieyu Gu^1^, Ran Yang^1^, Huailiang Huang^1^, Hongzhan Lin^1^, Liang Zhang^2,3^, Tao Zan^1^, Feng Xie^6*^, Kang Zhang^5*^ & Qingfeng Li^4*^

Correspondence to: [dr.liqingfeng@shsmu.edu.cn](mailto:dr.liqingfeng@shsmu.edu.cn) (Q.L.); [kang.zhang@gmail.com](mailto:kang.zhang@gmail.com) (K.Z.); [xiefenghe@163.com](mailto:xiefenghe@163.com) (F.X.)

**This PDF file includes:**

Figures. S1 to S10

Materials and Methods

**Figure S1. Most human and mouse GCMN cells are in a BCL2-addicted senescent state.**

**(a)** Immunohistochemistry staining (AEC) showing p-ERK, p-AKT, Ki67, and P16 expression in normal skin tissues. Scale bar=50μm.

**(b)** Immunocytochemistry of melanocytes stained for senescence-associated secretory phenotype biomarkers (IL-6, SPP1, CCL2, and CXCL8), senescence-associated heterochromatic foci (H3K9me2 and mH2A), and cell proliferation markers (Ki67 and P16). Scale bar=50μm.

**(c)** Immunohistochemistry staining (AEC) showing Lamin B1 expression in GCMN and normal skin tissues. Scale bar=50μm.

**(d)** Immunocytochemistry of primary GCMN cells and melanocytes stained for Lamin B1. Scale bar=50μm.

**(e-f)** Cell cycle analysis of primary GCMN cells (n=3) and primary melanocytes (n=3). Red, yellow, and green peaks represent G0/G1, S, and G2 phases, respectively. Mean ± SD.

**(g)** Correlation analysis between the proportion of P16+ senescent GCMN cells and age (n=36).

**(h)** RNA-seq analysis of GCMN tissues (n=4) and adjacent normal skin tissues (n=4). Differentially expressed genes were used for functional enrichment analysis. The top 10 downregulated gene ontology terms are listed according to the adjusted p-value, with the most downregulated term highlighted by a red frame.

**(i)** Immunohistochemistry staining (AEC) of GCMN tissue microarray showing extensive BCL2 expression across different genotypes. Scale bar=400μm.

**(j)** Flow cytometry analysis detecting SOX10+/BCL2+ cells in melanocytes and GCMN cells.

**(k)** Percentage of double-positive BCL2 and SOX10 cells in GCMN cells (n=3) and melanocytes (n=3), as detected by flow cytometry. Mean ± SD.

**(l)** Immunostaining detects GCMN biomarkers, senescent biomarkers, anti-apoptotic activity, growth arrest, and stem cell biomarkers in the skin tissues of H11-Tyr-*Nras*^Q61K^ mice. Scale bar=50μm or 100μm.

**Figure S2. Construction strategies for H11-*Tyr*-*Nras*^Q61K^ and H11-*Tyr*-*Braf*^V600E^ GCMN mice.**

**(a)** Diagram illustrating the targeting locus of *Hipp11* genes.

**(b)** Diagram showing the inserted targeting vector for H11-*Tyr*-LSL- *Nras*^Q61K^ mice. The H11-*Tyr*-LSL- *Nras*^Q61K^ mice are crossed with *Dppa3*-Cre mice to generate H11-*Tyr*- *Nras*^Q61K^ mice.

**(c)** PCR analysis confirming the successful construction of H11-*Tyr*- *Nras*^Q61K^.

**(d)** Diagram showing the inserted targeting vector for H11-*Tyr*-*Braf*^V600E^ mice.

**(e)** PCR analysis confirming the successful construction of H11-*Tyr*-*Braf*^V600E^ mice. Heterozygous mice are used for further experiments, as human GCMNs are caused by heterozygous *NRAS* or *BRAF* mutations.

**Figure S3. *Nras*^Q61K^-mutated transgenic GCMN mice recapitulate the phenotypes of human GCMN.**

**(a)** The distribution patterns of GCMN lesions in H11-*Tyr*- *Nras*^Q61K^ mice mirror the 6B distribution (bolero, back, bathing trunk, breast/belly, body extremity, and body) observed in human GCMN cases.

**(b)** HE staining revealing histological features and hyperpigmentation in the paw, tail, skin, and hair of H11-*Tyr*- *Nras*^Q61K^ mice. Scale bar=100μm.

**(c)** Immunostaining identifies MelanA+ GCMN cells in the paw, tail, skin, and hair follicles of H11-*Tyr*- *Nras*^Q61K^ mice. Scale bar=100μm.

**Figure S4. *Braf*^V600E^-mutated transgenic mice exhibit GCMN phenotypes, which regress after Venetoclax treatment.**

**(a)** Phenotypes of H11-*Tyr*- *Braf*^V600E^ and wild-type mice. The left panel shows wild-type mice, while the right panel shows H11-*Tyr*- *Braf*^V600E^ mice. Notable features in H11-*Tyr*- *Braf*^V600E^ mice include darker hair and hyperpigmentation of the paws, tail, and skin tissues.

**(b)** Phenotypes of *Braf*^V600E^ GCMN mice before and after intralesional injection of DMSO or Venetoclax.

**(c)** Leukotrichia phenotype observed in *Braf*^V600E^ mice 30 days after intralesional Venetoclax injection.

**(d)** HE staining demonstrating immune cell infiltration, reduced melanin and nevocytes following Venetoclax treatment. Scale bar=100μm.

**Figure S5. Anti-BCL2 therapy effectively eliminates GCMN cells with different genotypes while exhibiting relatively minimal cytotoxicity in normal melanocytes, fibroblasts, and keratinocytes.**

**(a-b)** Morphology of primary GCMN cells with different genotypes, primary normal melanocytes, fibroblasts, and keratinocytes after 72 hours of treatment with DMSO, Trametinib, and Venetoclax. Scale bar=100μm.

**(c)** CCK8 assay demonstrating the cell viability of normal melanocytes, fibroblasts, keratinocytes, and GCMN cells after 72 hours of treatment with different concentrations of Venetoclax.

**(d-e)** Flow cytometry analysis detecting apoptosis of keratinocytes, fibroblasts, normal melanocytes, and GCMN cells after 72 hours of treatment with DMSO, and Venetoclax (n=3). Mean ± SD.

**(f)** Morphology of co-culture Fibroblasts + GCMN and co-culture Keratinocytes + GCMN cells after 72 hours of treatment with DMSO and Venetoclax. Scale bar=100μm.

**(g)** HE staining and immunostaining labeling S100B+ cells demonstrating the efficacy of DMSO, Trametinib, and Venetoclax treatment in GCMN ex vivo explants after 5 days. Scale bar=50μm.

**(h)** Immunostaining labeling S100B+/MelanA+ and BCL2+ GCMN cells in GCMN tissues from PDX models following 7 days of intralesional injection with Venetoclax or DMSO. Scale bar=100μm.

**(i)** Quantitative PCR assessing the relative BCL2 expression in GCMN cells after siNC (n=3) and siBCL2 treatments (n=3). Mean ± SD.

**(j)** Morphology of GCMN cells following siNC and siBCL2 treatments. Scale bar=100μm.

**(k)** Flow cytometry analysis detecting apoptosis (PI and Annexin V expression) in GCMN cells after siNC and siBCL2 treatments.

**(l)** Cell-derived xenograft constructed with siNC and siBCL2 treated GCMN cells. A total of 5x10^6^ cells were injected intradermally and harvested after one month. The black arrow indicates the melanin produced by GCMN cells, signifying the successful model construction.

**Figure S6. BCL2 inhibitor induces grey hair in unshaved GCMN mice.**

**(a-c)** Visualization and dermoscopic images of tail and hair phenotypes following 14 days of intralesional injections with DMSO, Trametinib, or Venetoclax in unshaved H11-*Tyr*- *Nras*^Q61K^ mice.

**(d)** HE staining illustrating the histological changes in the tail and hair follicles after 14 days of intralesional injections with DMSO, Trametinib, or Venetoclax. Scale bar=100μm.

**(e)** Phenotypic evaluations of the hair in wild-type mice at 90 days post-treatment of intralesional injection with Venetoclax.

**(f-g)** Immunostaining labeling MelanA+ cells in the hair follicles of H11-*Tyr*- *Nras*^Q61K^ mice and wild-type mice following 14 days of intralesional injection with DMSO and Venetoclax (n=30 follicles for each group). Scale bar=50μm.

**Figure S7. Oral BCL2i treatment effectively regresses mouse GCMN lesions without causing acute organ injury.**

**(a)** Timeline illustrating the oral gavage administration of BCL2i in H11-*Tyr*-*Nras*^Q61K^ GCMN mice.

**(b)** Regression and leukotrichia observed in GCMN lesions following oral BCL2i treatment.

**(c)** HE staining showing reduced melanin, GCMN cells, and hair follicles in skin tissues after oral Venetoclax treatment. Scale bar=100μm.

**(d)** Melanin content analysis revealing significantly reduced melanin in lesions of the oral BCL2i-treated group (n=15 mouse for each group). Mean ± SD.

**(e)** HE staining showing no recurrence of lesions 335 days post-Venetoclax treatment, with significant immune cell infiltration observed in the dermis of regressed lesions. Scale bar=100μm.

**(f)** Immunostaining indicating that the majority of resident immune cells are S100A8/9+/IL1B+ neutrophils, with a minority being CD3+ T cells and CD20+ B cells. Scale bar=100μm.

**(g-h)** Histology and functional analysis of organs and blood cell count in Venetoclax-treated mice showed normal results compared to DMSO-treated mice, suggesting minimal organ toxicity and hematological suppression (n=3 mouse for each group). Scale bar=100μm. Mean ± SD.

**Figure S8. Cell communication analysis shows that neutrophils interact with stressed nevocytes via IL-1 signaling pathway.**

**(a)** The cell viability assays of cultured mouse GCMN cells underwent different concentrations of Venetoclax treatment in vitro.

**(b)** Plasma pharmacokinetics curve of GCMN mouse after Venetoclax intralesional injection (n=3 mouse for each time point). Mean ± SD.

**(c)** Violin plots illustrating marker gene expression across distinct cell clusters.

**(d)** tSNE visualization collectively illustrating cell clusters among three groups, including Venetoclax-treated GCMN, untreated GCMN, and normal skin.

**(e)** Composition of all cell clusters across the three groups.

**(f)** tSNE visualization collectively depicting melanocyte subclusters among the three groups.

**(g)** Composition of each melanocyte subcluster among the three groups.

**(h)** Violin plots demonstrating the expression of apoptosis-related genes among all melanocyte subclusters.

**(i)** Top 20 overexpressed genes in the neutrophil cluster versus the other clusters.

**(j)** Interaction number and strength of cell communication among the three groups.

**(k)** Pathways mediating cell communication among the three groups. The IL-1 signaling pathway is specifically shown in Venetoclax-treated GCMN, highlighted with a red frame.

**(l)** Recruited neutrophils exhibit strong interaction with stressed-state nevocytes via the IL-1 signaling pathway.

**(m)** Violin plots showing the expression of IL-1 ligands and IL-1 receptors among melanocyte clusters and neutrophils. Both IL-1 ligands and IL-1 receptors are not expressed in normal melanocytes, while IL-1 receptors are highly expressed in nevocyte clusters, highlighted with a red frame.

**(n)** Contribution of different ligand-receptor pairs in the IL-1 signaling pathway in BCL2i-treated GCMN.

**Figure S9. Single-cell sequencing and immunostaining reveal the emergence of proliferative and reparative keratinocytes in Venetoclax-treated mouse GCMN.**

**(a-b)** tSNE visualization depicting keratinocyte subclusters among all three groups, highlighting the presence of subcluster 0 as the predominant population in the treated nevi group.

**(c)** Violin plots displaying the expression of proliferative and reparative keratinocyte markers across the four keratinocyte subclusters.

**(d)** Violin plots showing the expression of cell growth genes among the keratinocytes of three groups.

**(e-f)** GO biological process and KEGG pathway enrichment analysis for cluster 0 of keratinocytes.

**(g)** Immunostaining showing KRT6A expression in Venetoclax-treated nevi, DMSO-treated nevi, Venetoclax-treated normal skin, and DMSO-treated normal skin from the mouse. Scale bar=25μm.

**(h)** Immunostaining demonstrating KRT6A expression in spontaneously regressed nevi, normal nevi, and normal skin from humans. Scale bar=100μm.

**Figure S10. Venetoclax-induced leukotrichia is reduced in mice with neutrophil depletion and IL-1β inhibition.**

**(a-b)** Flow cytometry analysis demonstrating the effectiveness of immune cell depletion and IL-1β inhibition in H11-*Tyr*-*Nras*^Q61K^ GCMN mice.

**(c)** Phenotypes of GCMN lesions exhibiting the retained efficacy of Venetoclax treatment in groups with T cell, B cell, NK cell, and macrophage depletion. The red frames indicate the areas of Venetoclax-injected GCMN lesions.

**(d)** Hair phenotypes observed 14 days post-Venetoclax injection across different experimental groups.

Supplementary Materials and Methods

**Total RNA extraction and RNA sequencing**

Universal RNA Purification Kit (EZB-RN4, EZBioscience) was used to extract total RNA from the cells or tissues according to the manufacturer’s instructions. The RNA concentration was quantified with a Qubit RNA BR Assay Kit (Q10210, Invitrogen), and the integrity of the RNA was assessed with a Bioanalyzer RNA 6000 Nano Kit (5067-1511, Agilent Technologies). Sequencing libraries were constructed using a Tru-Seq RNA Sample Preparation Kit (Illumina). Briefly, 500 ng of total RNA was enriched for the mRNA fraction using oligo-dT magnetic beads. The mRNA was fragmented using divalent metal cations at high temperature. Second-strand cDNA synthesis was subsequently performed. The blunt-ended double-stranded cDNA was 3’ adenylated, and Illumina-indexed adapters were ligated. The product was enriched with 15 PCR cycles, and the library was validated on an Agilent 2100 Bioanalyzer. The libraries were sequenced on a NovaSeq 6000 (Illumina) in paired-end mode. The sequencing reads were mapped to the STAR GRCh37 human reference genome. The differentially expressed genes (DEGs) were determined via the DESeq2 Bioconductor R package. A log ∣fold change∣ >2 and P value < 0.05 were considered significant in this study. GO and Kyoto Encyclopedia of Genes and Genomes (KEGG) pathway enrichment analyses were performed using the Gene Set Enrichment Analysis (GSEA) and Database for Annotation, Visualization, and Integrated Discovery (DAVID). A P value < 0.05 was considered significant.

**Single-cell RNA data analysis**

The raw data were processed using the CellRanger package (version 6.1.2) for initial metric assessment. Subsequently, the data were filtered according to the following criteria using Seurat (version 5.0.3): (1) cells with more than 1,000 unique molecular identifiers (UMIs); (2) cells expressing more than 200 genes; (3) a cell complexity of 0.7 (log10 genes per UMI); (4) cells with a mitochondrial gene ratio of less than 0.1; and (5) genes expressed in more than 3 cells. DoubletFinder (version 2.0) was utilized to remove doublet cells. Following quality control, 47,803 cells with 22,323 genes remained. Conventional data processing included the following steps: (1) normalization of gene expression matrices using the ‘LogNormalize’ method; (2) selection of the 2,000 most highly variable genes to standardize the matrices; and (3) execution of Principal Component Analysis (PCA) and t-distributed Stochastic Neighbor Embedding (t-SNE) to group the cells into 23 clusters (resolution = 0.4). Using the ‘runPrestoAll’ function from the SeuratWrappers package (version 0.3.1) with the Wilcoxon method, we identified differentially expressed genes (DEGs) with an adjusted P-value of less than 0.05 and a log fold change greater than 0.25. Based on the DEGs and the ACT website (https://xteam.xbio.top/ACT/), we conducted manual annotation for each cluster. Overrepresentation analysis (ORA), including Gene Ontology (GO) analysis of the DEGs, was performed using BioEnrichr (version 0.1.0). The reference databases included the Molecular Signatures Database (MSigDB), specifically the canonical pathways (CP) KEGG, CP REACTOME, and Hallmark. Additionally, irGSEA (version 3.2.2) was employed for 'Stress' scoring of melanocyte subclusters. Finally, we utilized CellChat (version 1.6.1) to analyze the interactions between subclusters of melanocytes and neutrophils mediated by known ligand-receptor pairs. This analysis provided further insights into the mechanisms of cytotoxicity towards GCMN mediated by neutrophils following BCL2 inhibitor intervention.

**Antibodies**

BCL2 (ab182858, Abcam), BCL2 (60178-1-Ig, Proteintech), BCL2 Alexa Fluor® 488 (sc-7382, Santa Cruz), S100B (ab52642, Abcam), S100B (66616-1-Ig, Proteintech), CDKN2A/p16INK4a (ab201980, Abcam), CDKN2A/p16INK4a (ab270058, Abcam), Ki67 (ab279653, Abcam), CD4 (A19018, Abclonal), CD3 epsilon (ab5690, Abcam), MelanA (GTX34832, Genetex), SOX10(GTX35085, Genetex), SOX10 (ab227680, Abcam), SOX10 Alexa Fluor® 647 (ab270151, Abcam), HMB45 (GTX71957, Genetex) , p44/42 MAPK (Erk1/2) (4695, CST), CD20 (60271-1-Ig, Proteintech), CD117/c-Kit (14-1171-82, Invitrogen), Nestin (ab316018, Abcam), Fascin (ab126772, Abcam), p-GSK3B (67558-1-Ig, Proteintech), SPP1 (sc-73631, Santa Cruz), CXCL8 (17038-1-AP, Proteintech), IL-1β (12242, CST), IL-1β CoraLite® Plus 488 (CL488-66737, Proteintech), IL-6 (21865-1-AP, Proteintech), IL-6 (ab233706, Abcam), MPO (ab300650, Abcam), S100A8 + S100A9 (ab288715, Abcam), MITF (ab303530, Abcam), MITF (PA5-99499, Invitrogen), TRP1(ab235447, Abcam), ELANE (ab314916, Abcam), Ly6G (14-5931-82, Invitrogen), Citrullinated Histone H3 (97272, CST), Cytokeratin 6A (19401-1-AP, Proteintech), CCL2 (14-7096-81, Invitrogen), Lamin B1 (F0523, Selleck), H3K9me2 (ab1220, Abcam), mH2A1 (ab183041, Abcam), CD4-APC (17-0042-82, Invitrogen), CD8-APC (17-0081-82, Invitrogen), CD19 (17-0193-82, Invitrogen), F4/80-PE (12-4801-82, Invitrogen), Ly6G-PE (12-9668-82, Invitrogen), NK1.1-PE (12-5941-82, Invitrogen).

HRP-labeled Goat Anti-Mouse IgG (H+L) (A0216, Beyotime), HRP-labeled Goat Anti-Rabbit IgG(H+L) (A0208, Beyotime), Goat Anti-mouse Alexa Fluor 488 (ab150113, Abcam), Goat Anti-mouse Alexa Fluor 647 (ab150115, Abcam), Goat Anti-rabbit Alexa 488 (ab150077, Abcam), Goat Anti-rabbit Alexa Fluor 647 (ab150079, Abcam), Goat anti-rat Alexa Fluor 488 (ab150157, Abcam), Goat Anti-Armenian Hamster Alexa Fluor 488 (ab173003, Abcam).
